# Lens-Induced Glaucoma: The Need to Spread Awareness about Early Management of Cataract among Rural Population

**DOI:** 10.1155/2013/581727

**Published:** 2013-06-25

**Authors:** Raghunandan Kothari, Sandeep Tathe, Pratik Gogri, Akshay Bhandari

**Affiliations:** Department of Ophthalmology, Rural Medical College of Pravara Institute of Medical Sciences, Loni, Taluka Rahata, District Ahmednagar, Maharashtra 413736, India

## Abstract

*Purpose*. To determine the clinical profile of lens-induced glaucoma (LIG), reasons for late presentation, and outcome of current management. *Methods*. Retrospective analysis of 50 eyes with LIG over a 6-year period between 2005 and 2011 at a tertiary care centre in rural India. Visual acuity and intraocular pressure (IOP) were assessed preoperatively and postoperatively along with postoperative complications. *Results*. Fifty (2.4%) of 12,004 senile cataracts operated at Pravara Rural Hospital, Loni, presented with LIG. There were 39 (78%) phacomorphic cases and 11 (22%) phacolytic glaucoma. Following cataract surgery, 21 of 50 operated eyes (42%) had visual acuity 6/60 or worse. *Conclusion*. The results highlight the importance of early diagnosis and treatment of visually disabling cataract. There is a need to educate both the patient and the cataract surgeon about the dangers of lens-induced glaucoma and of about poor outcome if treatment is delayed.

## 1. Introduction

There are twenty million blind people in India; eighty percent of this blindness is due to causes which are preventable. Cataract in India is the most important cause of preventable blindness accounting to 63.7 percent [1].

Lens-induced glaucoma (LIG) was first described in the year 1900 by Gifford [2] and von Reuss [3] independent of each other. While the former described it as a glaucoma associated with hypermature cataract, the latter described it as a glaucoma associated with spontaneous absorption of lens substance through intact lens capsule. Subsequently, various workers [4–6] described such types of cases under different names like LIG, lens-induced uveitis and glaucoma, phacotoxic glaucoma, phacogenic glaucoma, and finally phacolytic glaucoma. These terms including the more popular term phacolytic glaucoma have been discarded for various reasons and convenience in favour of the term “LIG.” At present, LIG is a clinical condition characterised by (i) a violent secondary glaucoma (resembling acute angle closure glaucoma) in one eye with senile mature cataract, hyper mature senile cataract (rarely immature senile cataract) yet with an open angle, (ii) normal intraocular pressure and open angle in other eye, and (iii) a prompt relief of symptoms and restoration of vision after cataract extraction in the effected eye.

Late reporting for treatment of cataract leading to serious complications like LIG remains one of the most important cause of irreversible loss of vision, especially so in the rural population. This preventable and curable condition, though rare in developed countries, is unfortunately still prevalent in India.

The study is undertaken to study the profile of lens-induced glaucoma leading to blindness in rural population in western Maharashtra, India. It is a retrospective hospital-based study. The records of cataract cases who were diagnosed as LIG and operated at our hospital from 2005 to 2011 were analyzed.

## 2. Material and Methods

Fifty cases included in this study were operated for lens-induced glaucoma between 2005 and 2011 at Pravara Rural Hospital, Loni, Maharashtra, India. All of them reported with pain, redness, and watering of acute onset in addition to gradual progressive loss of vision in affected eye as their presenting complaints and were diagnosed as LIG based on the clinical findings and raised intraocular pressure. Congenital cataract, secondary cataract, traumatic cataract, complicated cataract, and known cases of glaucoma were excluded from the study. Complete ophthalmic examination with preoperative and postoperative visual acuity, IOP measurement, slit lamp examination, and intraoperative and postoperative complications were recorded.

## 3. Results

Fifty cases recorded over 6 years contributed about 2.4 percent of all cases of senile cataract admitted for cataract extraction during this period. There were 18 males and 32 females. Their age ranged from 56 to 81 years with a mean of 68.84 years. The youngest patient was a 56-year-old female and the oldest an 81-year-old male. Females outnumbered the males 1.8 : 1. Age- and sex-wise distribution is as shown in Table 1. Cases were diagnosed as phacomorphic based on presence of pain and redness of the affected eye associated with the presence of corneal oedema, shallow anterior chamber, dilated pupil, intumescent cataractous lens, and intraocular pressure above 21 mm Hg. Eleven cases were diagnosed as phacolytic glaucoma. No cases were reported of phacoanaphylactic glaucoma. Symptoms of pain and redness improved in the majority of cases; however, there was no remarkable improvement in visual acuity after cataract surgery in most cases.

The time gap between onset of acute symptoms of pain, redness, marked reduction of vision, and reporting of patients to hospital is as shown in Table 2. Eight cases (16%) presented to the hospital within 48 hours of onset of the disease. Twenty-two patients (44%) came to the hospital between the 3rd and the 6th day. In twenty patients (40%), the disease had been present for more than a week before any treatment was sought.

The visual acuity was markedly reduced in all cases due to cataract as well as due to loss of corneal transparency secondary to a sudden rise of intraocular pressure. Thirty-nine patients had only light perception, and in three cases, even perception of light was doubtful. The mean preoperative intraocular pressure was 44 mm Hg (range 24–68). In 25 (61%) patients, the intraocular pressure could be reduced to less than 30 mm Hg by hypotensive medications.

The postoperative vision of patients was as shown in Table 3. It can be seen that 16% of the patients recovered very good vision (6/18 or better) after surgery. Low vision/visual impairment (<6/18–6/60) occurred in 12 (24%) cases. Blindness (<6/60–PL+) occurred in 28 (56%) cases. Total blindness (no PL) occurred in 2 (4%) cases (Table 3).

Intraoperative and postoperative complications encountered in this study are as shown in Table 4. While performing the surgery, shallow anterior chamber due to posterior vitreous pressure was seen in 12 patients. Posterior capsular tear resulting in loss of the vitreous occurred in five patients, and in three patients, complete removal of the cortical material could not be achieved. Postoperative uveitis was seen in 19 (33.3%) patients and striate keratopathy in 14 (24.6%) cases. The rates of other complications are as shown in Table 4.

## 4. Discussion

Cataract remains the most important cause of blindness in developing countries, affecting mostly the older rural population. Delayed reporting for treatment leads to serious complications like lens-induced glaucoma causing irreversible visual loss. LIG is a condition to reckon with in our ophthalmic patients, especially ours being a rural area. In spite of easy availability of surgical facilities with concerted efforts of the National Programme for Control of Blindness (NPCB), NGOs, government agencies, and private practitioners, cataract surgery being a very cost effective and rewarding surgery, still many people are becoming blind due to lack of awareness about significance of early management. Illiterate, older, and rural population are the worst affected. In this study, 30 patients got no visual improvement or only marginal improvement in vision after the operation. This was the group where time lag between development of symptoms of pain/redness and reporting for treatment was the longest. Visual outcome after cataract surgery has been reported normal, that is, 6/18 or better only in 16 percent eyes, and blindness (<6/60–PL+) occurred in 28 (56%) cases. 45.7 percent of eyes with presenting vision of less than 3/60 after cataract surgery could not be improved with correction (NPCB). The most important cause of poor postoperative vision has been attributed to surgical complications [7]; however, in our study, late reporting for treatment also emerged as one of the most important causes for poor postoperative vision following cataract surgery. Females have been reported to have more adverse outcome (36.2 percent) as compared to males (30.5%) [8].

In our study, 60 percent had postoperative vision less than 6/60, which is quite less as compared to only 21 percent reported by Pradhan et al. [9]. However, in our study, no significant difference was found. The reason for delayed reporting in spite of services for cataract surgery available so easily appears to be poor health education, acceptance of poor vision as part of aging, fear of operation, lesser expectations and socioeconomic constraints. Also many people especially in rural areas take treatment for redness and pain in eyes from some local practitioners who miss the diagnosis initially. It was only when the symptoms became worse, they reported to the hospital. Another factor about late reporting found was that the very elderly visually handicapped persons were left to their own fate as no one bothered to bring them to the hospital. Presenting vision of most of the patients was less than 3/60 in our study. Presenting vision of less than 6/60 was reported by Murthy et al. [10] in a rural population, and poor visual outcome was reported to be due to surgery-related complications.

## 5. Conclusion

It is highlighted from our study that lack of awareness in a rural population about early treatment of cataract is a major detrimental factor in the visual outcome after cataract surgery, inspite of the advances in cataract surgery techniques. The present analysis of 50 cases of lens-induced glaucoma who received intraocular lens implants has shown that visual recovery and control of IOP following surgerywas not satisfactory due to late presentation of the majority of cases.

## Figures and Tables

**Table 1:** Age and sex distribution of patients.

Age (Yrs)	Male		Female		Total	
	Number of patients	%	Number of patients	%	Number of patients	%
51–60	2	4	1	2	3	6
61–70	9	18	24	48	33	66
71–80	6	12	7	14	13	26
More than 80	1	2	0	0	1	2

Total	18	36	32	64	50	100

**Table 2:** Duration of symptoms of lens-induced glaucoma in days.

Days	Number of patients	%
1	1	2
2	7	14
3	13	26
4	1	2
5	6	12
6	2	4
7	1	2
8 to 10	5	10
11 to 14	0	0
More than 14	14	28

**Table 3:** Postoperative visual acuity of patients.

Vision	Number of patients	Percent
6/6–6/18	8	16
<6/18–6/60	12	24
<6/60–3/60	9	18
<3/60–1/60	12	24
<1/60–PL	7	14
No PL	2	4

Total	50	100

**Table 4:** Intraoperative and postoperative cataract surgery complications.

Intraoperative and postoperative complications	Cases	%
Shallow anterior chamber	12	21.1
Posterior capsule rupture and vitreous loss	05	8.7
Cortical remnants	03	5.3
Hyphema	04	7
Anterior uveitis	19	33.3
Striate keratopathy	14	24.6
